# Transcriptome analysis provides new ideas for studying the regulation of glucose-induced lignin biosynthesis in pear calli

**DOI:** 10.1186/s12870-022-03658-x

**Published:** 2022-06-27

**Authors:** Yuru Jiao, Xin Gong, Kaijie Qi, Zhihua Xie, Yanling Wang, Kaili Yuan, Qi Pan, Shaoling Zhang, Katsuhiro Shiratake, Shahrokh Khanizadeh, Shutian Tao

**Affiliations:** 1 College of Horticulture, Nanjing Agricultural University, Nanjing, China; 2 State Key Laboratory of Crop Genetics and Germplasm Enhancement, Nanjing Agricultural University, Nanjing, China; 3 Laboratory of Horticultural Science, Nagoya University, Nagoya, Japan; 4 Elm Consulting, Genetic Research, St-Lazare, Quebec, J7T 3C2 Canada

**Keywords:** Pear calli, Transcriptome analysis, Glucose treatment, Lignin

## Abstract

**Background:**

Glucose can be involved in metabolic activities as a structural substance or signaling molecule and plays an important regulatory role in fruit development. Glucose metabolism is closely related to the phenylpropanoid pathway, but the specific role of glucose in regulating lignin biosynthesis in pear fruit is still unclear. The transcriptome of pear calli generated from fruit and treated with glucose was analyzed to investigate the role of glucose in lignin biosynthesis.

**Results:**

The treatment of exogenous glucose significantly enhanced the accumulation of lignin in pear calli. A total of 6566 differentially expressed genes were obtained by transcriptome sequencing. Glycolysis was found to be the pathway with significant changes. Many differentially expressed genes were enriched in secondary metabolic pathways, especially the phenylpropanoid pathway. Expression of structural genes (*PbPAL*, *PbHCT*, *PbCOMT*, *PbPRX*) in lignin biosynthesis was up-regulated after glucose treatment. In addition, glucose might regulate lignin biosynthesis through interactions with ABA, GA, and SA signaling. Several candidate *MYB* transcription factors involved in glucose-induced lignin biosynthesis have also been revealed. The qRT-PCR analyses showed that the expression pattern of *PbPFP* at early developmental stage in ‘Dangshansuli’ fruits was consistent with the trend of lignin content. Transient expression of *PbPFP* resulted in a significant increase of lignin content in ‘Dangshansuli’ fruits at 35 days after full bloom (DAB) and tobacco leaves, indicating that *PbPFP* (*Pbr015118.1*) might be associated with the enhancement of lignin biosynthesis in response to glucose treatment.

**Conclusions:**

*PbPFP* plays a positive role in regulating lignin biosynthesis in response to glucose treatment. This study may reveal the regulatory pathway related to lignin accumulation in pear calli induced by glucose.

**Supplementary Information:**

The online version contains supplementary material available at 10.1186/s12870-022-03658-x.

## Background

Lignin is a complex aromatic polymer and one of the important components of the secondary cell walls in plants [1]. In vascular plants, lignin provides structural rigidity and mechanical support, also promotes water transport [2]. Pear is one of the important fruit trees in China, and its planting area and yield of pears rank as the top of the world [3]. However, the significant accumulation of lignin in pear fruits could lead to a decrease in fruit quality with lower taste and economic value, as the formation of stone cells [4–6]. Therefore, reducing the content of lignin is an effective method to improve the quality of pear fruits.

As previous studies, sugar metabolism exists in the whole process of fruit development and is an important factor affecting the intrinsic quality of fruit [7–10]. There is a close relationship between lignin biosynthesis and sugar metabolism in plants. Specifically, lignin biosynthesis is linked to sugar metabolism through the shikimic acid pathway [11, 12]. In pears, the main photosynthetic products are sorbitol and sucrose, which are transported to sink tissues for metabolic activities or converted into other sugar components by enzymes [13]. The conversion of sucrose and sorbitol to glucose and fructose is crucial in carbon partitioning regulation [14]. The sucrose in the cytoplasm can be converted into fructose and glucose and transported to the vacuole. Sorbitol can also be converted into fructose and glucose by the catalysis of sorbitol dehydrogenase (NAD-SDH and NADP-SDH) and sorbitol oxidase (SOX) [15]. Then, they are phosphorylated into fructose 6-phosphate and glucose 6-phosphate by fructokinase and hexokinase, respectively. Fructose 6-phosphate and glucose 6-phosphate will engage in glycolysis and the pentose phosphate pathway (PPP) [16]. Their intermediates, 4-phosphate erythrose (E-4-P) and phosphoenolpyruvate (PEP), will be introduced into shikimate acid pathway for the synthesis of phenylalanine, which is well studied as one of the lignin precursors [17, 18]. Moreover, it is repoted that glycolysis could be activated along with activation of the phenylpropanoid pathway [19, 20]. Thus, there might be a link between sugar metabolism and the phenylpropanoid pathway, and even lignin biosynthesis.

Some research showed that carbon source of lignin biosynthesis was provided by glucose [21]. After D-glucose labeled with C^14^ was introduced into a spruce twig by infusion, lignin in the cambium was radioactive, indicating the ultimate organic source of lignin was D-glucose [22]. The content of lignin is also positively correlated with the protein level of pyruvate dehydrogenase (PDH E1α) [23]. It provides the potential relationship between two metabolic pathways about aerobic oxidation of glucose: glycolysis and tricarboxylic acid cycle [24]. In summary, sugar, especially glucose, is closely related to the biosynthesis of lignin.

In addition, the regulatory effects of glucose are usually accompanied by interactions with plant hormone signal transduction pathways. Accumulation of abscisic acid (ABA) and the expression of related genes could be significantly increased by glucose [25]. For example, ABA can significantly promote the granulation of the juice sac of grapefruit [26]. The lignin biosynthesis pathway is inhibited by gibberellin (GA) [27], while glucose can inhibits GA signaling at the late stages of the hormone transduction pathway [28]. Ethylene is an antagonist of glucose response [25], after treating with high concentration of glucose, *Arabidopsis* seedlings stopped growing, while the effect can be reversed with exogenous ethylene [29, 30]. Simultaneously, ethylene affects glucose signaling by reducing ABA biosynthesis, promoting germination and seedling development [30].

Moreover, the deposition of lignin occurs after the peak of sugar accumulation, and the amount of sugar accumulation directly affects the biosynthesis of lignin in pear fruits [31]. Exogenous glucose treatment promotes lignin deposition in *Arabidopsis* seedlings [32]. Sugar accumulation, especially glucose, also affects the lignin biosynthesis in peach [31]. However, the regulating mechanism of glucose on lignin biosynthesis in pear fruits is unclear. In this study, pear calli generated from the fruit was treated with exogenous glucose, and then employed to conduct a comprehensive transcriptome analysis to determine the differentially expressed genes (DEGs) between the glucose-treated and untreated control calli. Some important candidate genes associated with glucose metabolism and the phenylpropanoid pathway were identified to provide a valuable resource for further understanding the role of glucose in lignin biosynthesis.

## Results

### Determination of lignin content in calli of pear fruits

**Fig. 1:**
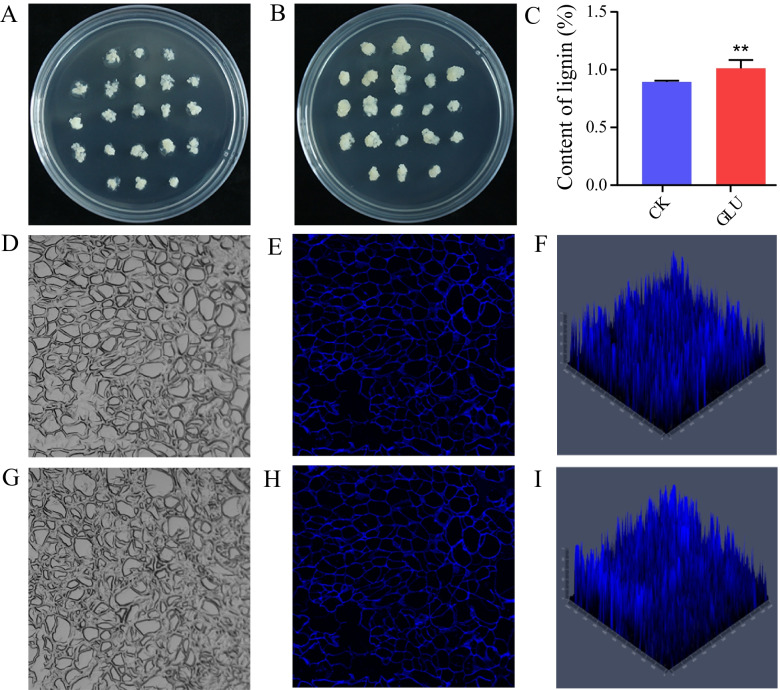
Glucose treatment significantly increased the lignin content of calli. **A** Calli treated with 0% glucose (CK). **B** Calli treated with 4% glucose (GLU). **C** The lignin contents of CK and GLU. **D** cell outline of CK. **E** and **F** Lignin autofluorescence of CK. **G** cell outline of GLU. **H** and **I** Lignin autofluorescence of GLU

In order to identify the effect of glucose on lignin biosynthesis in pear flesh calli, the calli were transferred to MS solid medium with different concentrations of glucose (0, 1, 2, 3, 4%), and the samples were collected at 15 d post-treatment. Lignin content of calli was significantly increased after treating with 1–4% glucose (Additional file 1: Fig. S1), and the difference in lignin contents between the calli treated with 4% glucose and the control was the most significant (Fig. 1C, Additional file 1: Fig. S1). The calli treated with 4% glucose grew faster, which was reflected in the larger volume (Fig. 1A and B), and the autofluorescence intensity of lignin was higher (Fig. 1D-I).

### Transcriptome sequencing

**Fig. 2:**
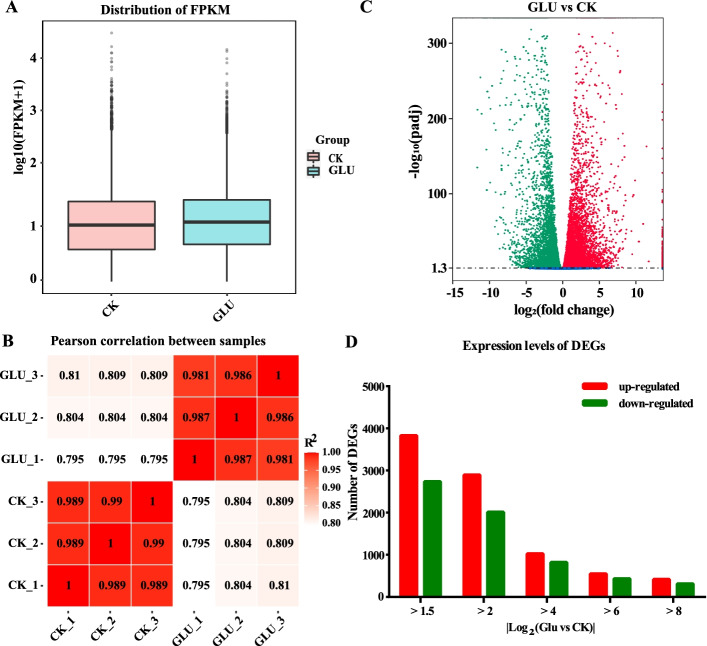
Analysis of the differentially expressed genes (DEGs). **A** Gene expression level boxplot. Each box plot corresponds to 5 statistics (from top to bottom are the maximum, upper quartile, median, lower quartile and minimum). **B** RNA-Seq correlation analysis. The closer the correlation coefficient is to 1, the higher the similarity of the expression patterns between samples. **C** Volcano plot of differentially expressed genes. The abscissa represents the fold change of gene expression in different samples, and the ordinate represents the statistical significance of the difference in gene expression; Genes with significant differential expression are represented by red dots (up-regulated) and green dots (down-regulated). Genes that are not significantly differentially expressed are indicated by blue dots. **D** Distribution of differentlly expressed genes

We used the calli treated with 4% glucose (GLU) and the control (CK) for transcriptome sequencing, then evaluated the quality of six cDNA libraries. The number of clean reads in each library ranged from 44 to 58 million, with an average Q20 (sequencing base calls with an error rate of < 1%) of > 96% (Additional file 4: Table S1) and a mapping rate of 79.38–82% (Additional file 5: Table S2). Density distribution profiles of fragments per kilobase of transcript per million mapped reads (FPKM) were constructed to reflect the gene expression pattern of each sample. We used log10-transformed data for further analysis, and the median gene expression levels of CK and GLU were around 1 (Fig. 2A). The Pearson correlation coefficient from the biological replicates of the same treatment varied from 0.981 to 0.99, showing that the biological reproducibility among the samples was good (Fig. 2B). These results indicated that data of transcriptome sequencing was reliable.

### Analysis of the differentially expressed genes (DEGs)

The DEGs were filtered by the following criteria: adjusted *p*-value < 0.05 and |log_2_(GLU vs CK)| ≥ 1.5. A total of 6566 DEGs were identified with 3829 up-regulated and 2737 down-regulated genes (Fig. 2C). When |log_2_(GLU vs CK)| ≥ 4, there were 1843 DEGs with 1025 up-regulated and 818 down-regulated genes. Moreover, when |log_2_(GLU vs CK)| ≥ 8, there were 723 DEGs with 413 up-regulated and 310 down-regulated genes (Fig. 2D).

### Functional analysis of DEGs

**Fig. 3:**
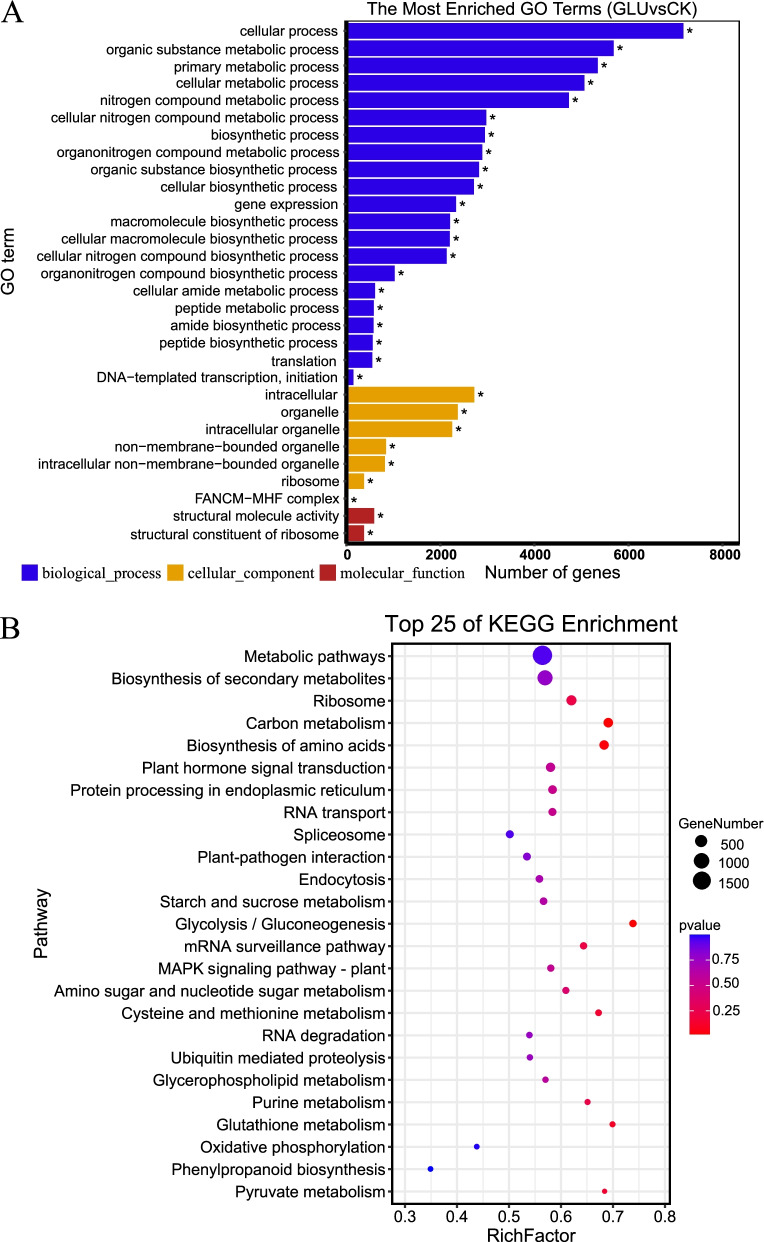
**A** GO annotation analysis of differentially expressed genes. **B** KEGG analysis of differentially expressed genes. The y-axis and x-axis present the KEGG pathways and the rich factors, respectively. Dot size corresponds to the number of distinct genes, whereas dot color reflects the q-value

Gene Ontology (GO) analysis was used to investigate the main functions of these specifically regulated genes. There were many DEGs related to biological processes and cellular components under glucose treatment. In the biological process category, most DEGs were associated with ‘cellular process’, ‘organic substance metabolic process’ and ‘primary metabolic process’. In the cellular component ontology, most DEGs were related to ‘intracellular’, ‘organelle’ and ‘intracellular organelle’. The GO term related to molecular function was significantly enriched in ‘structural molecule activity’ and ‘structural constituent of ribosome’ (Fig. 3A). The results provide a comprehensive perspective for screening candidate genes related to the enhancement of lignin biosynthesis in response to glucose treatment.

Then, the Kyoto Encyclopedia of Genes and Genomes (KEGG) database was used to analyze various metabolic pathways of DEGs to provide information and further understanding of how glucose regulates metabolism in cells and the synthesis of secondary metabolites, such as lignin, at the molecular level. We selected the top 25 KEGG enrichment pathways for analysis, including the carbon metabolism (250 DEGs), the plant hormone signal transduction (229 DEGs), glycolysis/gluconeogenesis (126 DEGs), mRNA surveillance pathway (122 DEGs) and phenylpropanoid biosynthesis (86 DEGs). Moreover, the expressions of genes involved in phenylalanine, tyrosine and tryptophan biosynthesis also changed (Fig. 3B).

#### DEGs involved in glycolysis/gluconeogenesis and the pentose phosphate pathway

**Fig. 4:**
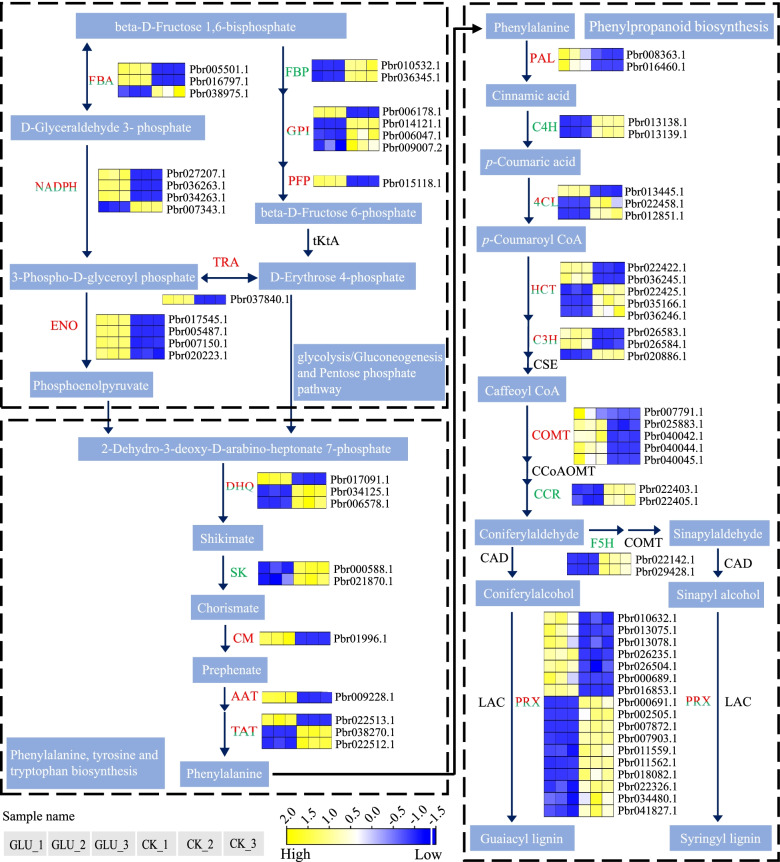
Transcriptional profiles of DEGs associated with Glycolysis/Gluconeogenesis and the pentose phosphate pathway, the phenylalanine, tyrosine and tryptophan biosynthesis, the phenylpropanoid biosynthesis pathway. The progression of the color scale from blue to yellow represents an increase in the FPKM values

Sugar provides energy for metabolism and signaling molecules for plants [33]. The genes involved in response to glucose were mainly related to glycolysis/gluconeogenesis and PPP (Fig. 4). Glycolysis is the core of carbon distribution of PPP, the shikimic acid pathway and the phenylpropanoid pathway [34]. The fine control of glycolysis has been reported to be exerted by hexokinase (HXK), phosphofructokinase (PFK) and pyruvate kinase (PK) [35, 36]. In glucose metabolism, no DEG was annotated as *PbHXK*, and three DEGs were annotated as *PbPK* (Pbr012277.1, Pbr000777.1, Pbr025447.1), whose expressions were up-regulated by glucose. Nevertheless, many DEGs were annotated into the PFK gene family, which had the largest number of DEGs in glycolysis, including *PbPFK* (6) and *PbPFP* (1) (Additional file 6: Table S3). The expression of 5 *PbPFK* (*Pbr008761.1*, *Pbr028610.1*, *Pbr028605.1*, *Pbr028606.1*, *Pbr028609.1*) and 1 *PbPFP* (Pbr015118.1) were all up-regulated in glucose-treated calli, suggesting that they might be the candidate genes for lignin biosynthesis enhancement in response to glucose.

#### DEGs involved in phenylpropanoid biosynthesis

The primary metabolism plays an important role in regulating secondary metabolism [37], and flux through primary metabolic pathways affects the flux into phenylpropanoid metabolism [38]. After RNA-seq analysis, the DEGs involved in phenylpropanoid biosynthesis were identified, and many genes were up-regulated (Fig. 4). The *PbPAL* (*Pbr008363.1* and *Pbr016460.1*), *PbCOMT* (*Pbr00779.1*, *Pbr025883.1*, *Pbr040042.1*, *Pbr040044.1*, *Pbr040045.1*) and some of *Pb4CL* (*Pbr013445.1*), *PbHCT* (*Pbr022422.1*, *Pbr036245.1*), *PbC3H* (*Pbr026583.1*, *Pbr026584.1*), *PbPRX* (*Pbr010632.1*, *Pbr013075.1*, *Pbr013078.1*, *Pbr026235.1*, etc.) were positively correlated with lignin biosynthesis in pear calli, and their expressions were significantly up-regulated under glucose treatment. However, the expressions of *PbC4H* (*Pbr013138.1*, *Pbr013139.1*), *PbCCR* (*Pbr022403.1*, *Pbr022405.1*), *PbF5H* (*Pbr022142.1*, *Pbr029428.1*) were down-regulated under glucose treatment. Nevertheless, these results indicated the activation of the phenylpropanoid pathway.

### Changes of transcription factors (TFs) under glucose treatment

**Fig. 5:**
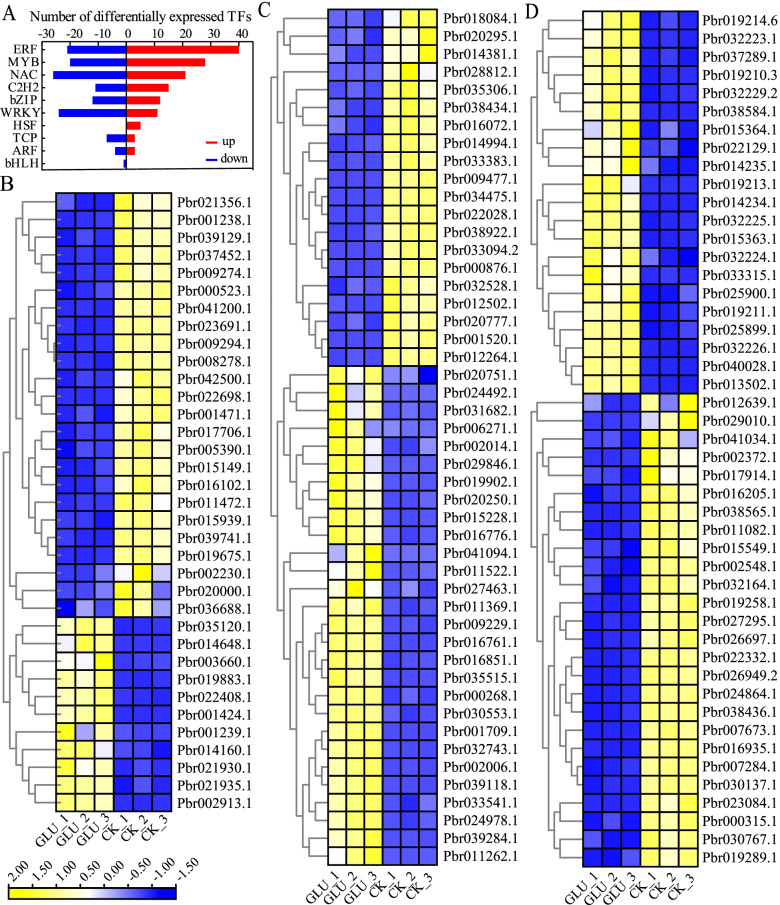
**A** Histogram of differentially expressed transcription factors. **B-D** Heat map of *PbWRKY* (**B**), *PbMYB* (**C**) and *PbNAC* (**D**) transcription factors expression

TFs are protein molecules with unique structures that can regulate genes to participate in various biological processes. *PbMYB* (48), *PbNAC* (47), and *PbWRKY* (35) were the most enriched TFs in this study (Fig. 5). The loss of functional *MYB103* resulted in changes in lignin monomer composition [39]. *Pbr035306.1* and *Pbr020777.1* were annotated as homologous genes of *AtMYB103*, and down-regulated in calli treated with glucose. *MYB52* could induce the expression of genes in the secondary wall biosynthesis pathway [40], *Pbr016851.1*, *Pbr002006.1* and *Pbr035515.1*, which are homologous genes of *AtMYB52*, were up-regulated by glucose. In addition, there were other TFs also identified, such as *PbERF*, *PbbHLH*, *PbbZIP*, *PbARF*, *PbC2H2*, *PbHSF* and *PbTCP* (Additional file 2: Fig. S2). The expression patterns of DEGs in these 10 TFs families were diverse. The DEGs in *PbC2H2*, *PbERF* and *PbMYB* families were mostly up-regulated, while in *PbWRKY*, *PbARF*, *PbNAC* and *PbTCP* families were mostly down-regulated. The analysis of TFs revealed that transcriptional effects are essential in the enhancement of lignin biosynthesis in response to glucose treatment.

#### DEGs involved in hormone signaling transduction

**Fig. 6:**
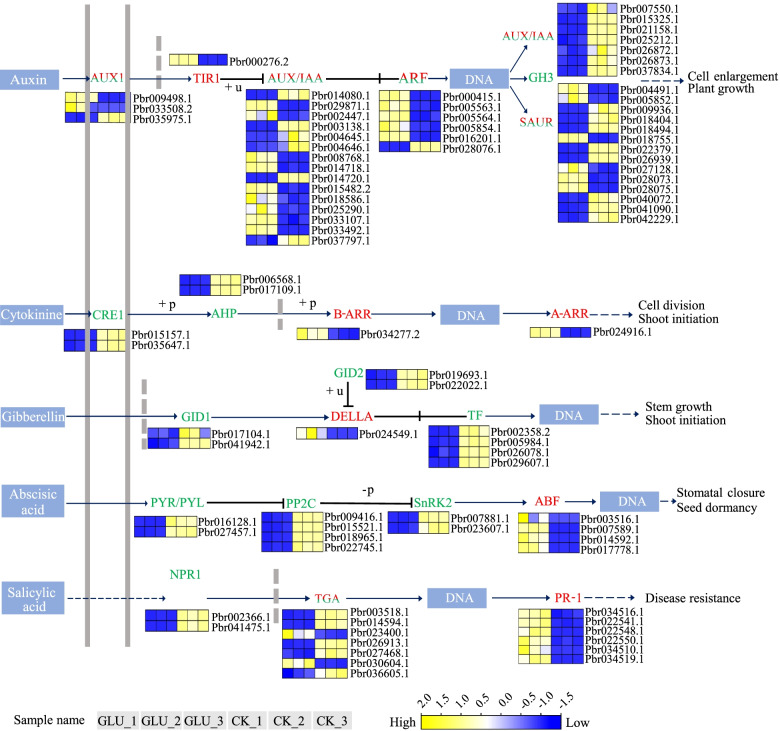
Transcriptional profiles of DEGs associated with the hormone signaling transduction. The progression of the color scale from blue to yellow represents an increase in the FPKM values

Plant hormones are essential components of the sugar sensing and signaling network that control plant growth and development [41, 42]. DEGs involved in hormone signaling pathways were enriched into eight groups, including auxin (46), cytokinin (6), GA (9), ABA (12), ethylene (8), brassinosteroid (5), jasmonic acid (6), and salicylic acid (SA) (15) signaling pathways (Additional file 7: Table S4). The number of DEGs in the auxin signaling pathway was more than in other hormone signaling pathways. 46 genes were annotated into the auxin signaling pathway and enriched into 6 families (*PbAUX1*, *PbTIR1*, *PbARF*, *PbAUX/IAA*, *PbGH3* and *PbSAUR*) (Fig. 6). Among them, the *PbAUX1* (2), *PbTIR1* (1), *PbAUX/IAA* (9), *PbARF* (5) and *PbSAUR* (6) genes were up-regulated after glucose treatment. ABA signaling is associated with plant secondary cell wall formation and lignin deposition [43]. In ABA signaling, the Log_2_ (GLU vs CK) fold changes of *PbABF* genes were more than 6, *PbPP2C* and *PbSnRK2* genes were on the contrary. GA regulates lignin biosynthesis [44]. A total of 9 genes from 3 families were enriched in the GA signaling pathway. In addition to the significant up-regulation of *PbDELLA* (*Pbr024549.1*), other genes were down-regulated. Moreover, six DEGs of the *PbPR-1* family were all up-regulated after glucose treatment in the SA signaling pathway.

### Validation of the expression patterns of selected DEGs by qRT-PCR

**Fig. 7:**
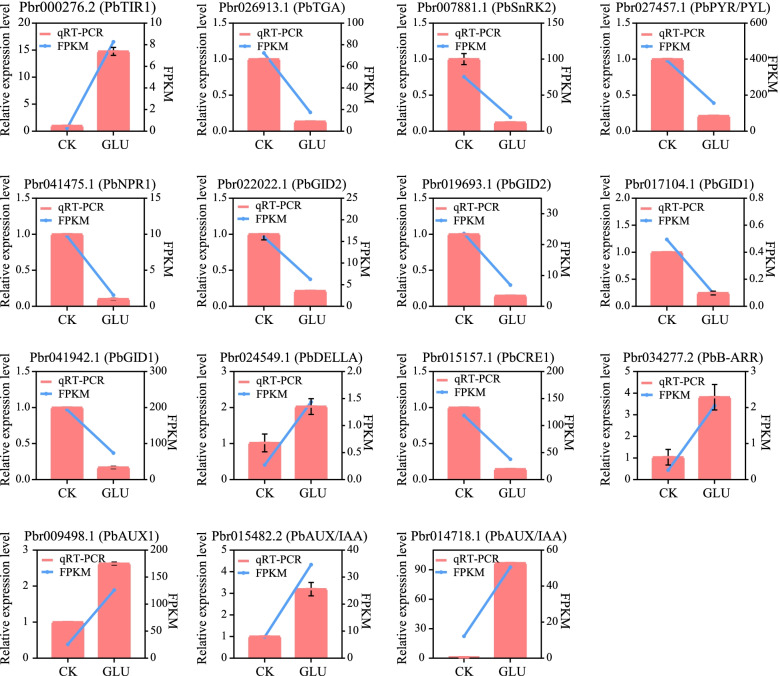
Validation by qRT-PCR of the expression patterns of DEGs of the hormone signaling transduction pathway. The right y-axis represents the selected gene expression levels calculated by the fragments per kilobase per million reads method, and the left y-axis represents the relative gene expression levels as assessed by qRT-PCR

**Fig. 8:**
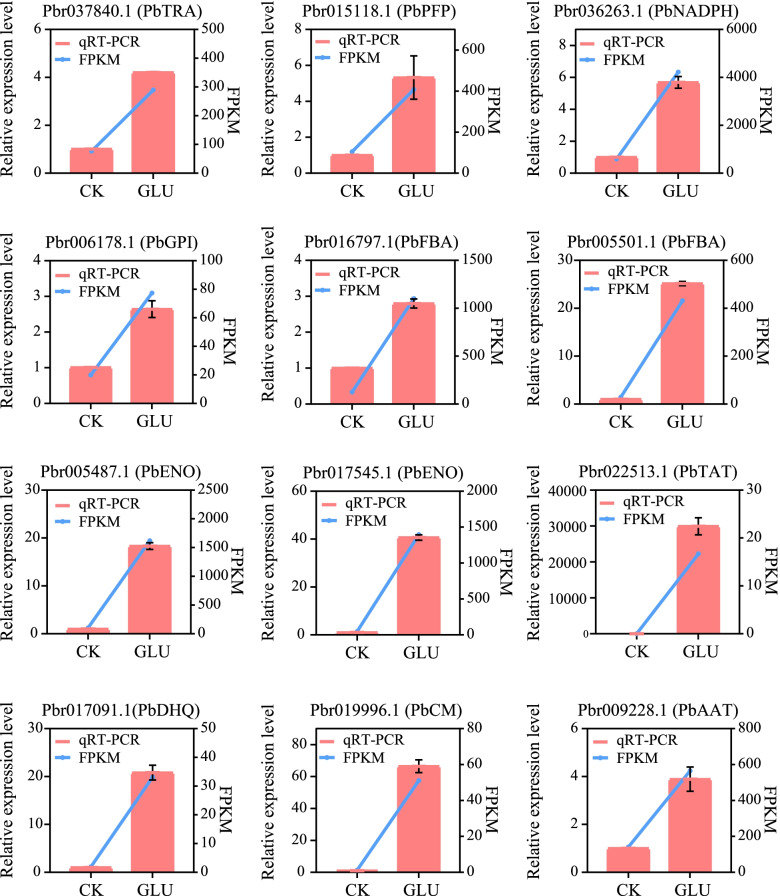
Validation by qRT-PCR of the expression patterns of DEGs of glycolysis/gluconeogenesis and the pentose phosphate pathway, the phenylalanine, tyrosine and tryptophan biosynthesis pathway. The right y-axis represents the selected gene expression levels calculated by the fragments per kilobase per million reads method, and the left y-axis represents the relative gene expression levels as assessed by qRT-PCR

**Fig. 9:**
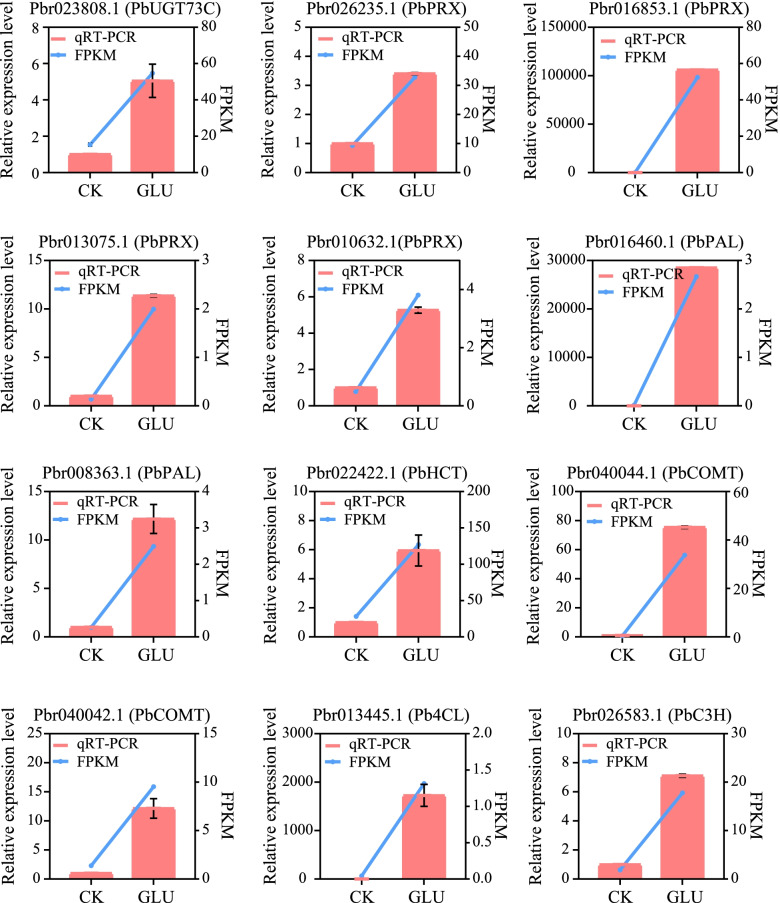
Validation by qRT-PCR of the expression patterns of DEGs of the phenylpropanoid pathway. The right y-axis represents the selected gene expression levels calculated by the fragments per kilobase per million reads method, and the left y-axis represents the relative gene expression levels as assessed by qRT-PCR

To verify the reliability of transcriptome analysis results, 39 DEGs were selected for qRT-PCR (Figs. 7, 8 and 9). They are involved in the hormone signaling pathway (Fig. 7), glycolysis/gluconeogenesis and the pentose phosphate pathway, the phenylalanine, tyrosine and the tryptophan biosynthesis pathway (Fig. 8), and phenylpropanoid biosynthesis pathway (Fig. 9). The expression trends of DEGs were largely consistent in the transcriptome and qRT-PCR, although the fold changes were different. Additionally, the correlation coefficient was 0.8561, indicating a positive correlation between RNA-seq and qRT-PCR data (Additional file 3: Fig. S3).

### Content of lignin significantly increased in pear fruit and tobacco leaves under transient expression of *PbPFP*

**Fig. 10:**
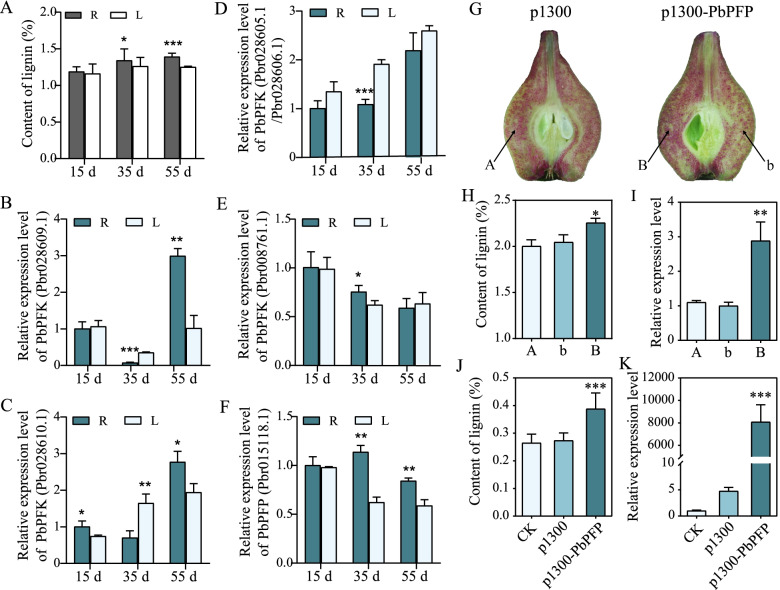
The content of lignin significantly increased after transient expression of *PbPFP* in pear fruit and tobacco (*Nicotiana tabacum* L.) leaves. **A** Lignin content in the area with more stone cells (R) and fewer stone cells (L) of ‘Dangshansuli’ fruits (15, 35, 55 days after full bloom). **B-F** Expression patterns of candidate genes. **G** Transient expression of *PbPFP* in ‘Dangshansuli’ fruits at 35 DAB. The infiltration sites were labelled A and B for the different gene constructs, and the non-infiltrated site was labelled b. **H** The content of lignin in pear fruits. **I** The expression patterns of *PbPFP* in pear fruits. **J** The content of lignin in tobacco leaves. **K** The expression patterns of *PbPFP* in tobacco leaves

The activation of glycolysis is closely related to the increased biosynthesis of lignin in the phenylpropanoid pathway [19, 20, 45]. To investigate the role of primary metabolism in lignin biosynthesis under glucose treatment, the candidate genes in glycolysis were selected for further analysis. The expression patterns of six candidate genes at early fruit developmental stages as 15, 35, 55 DAB in ‘Dangshansuli’ fruits were studied by qRT-PCR. It was found that the expression trend of *PbPFP* at the three stages was most consistent with the trend of lignin content, and the expression level of *PbPFP* was higher in the area with more stone cells (R) than in the area with fewer stone cells (L) (Fig. 10A-F). Therefore, it was speculated that *PbPFP* might be associated with the enhancement of lignin biosynthesis in response to glucose treatment.

To further investigate the role of PFP in lignin biosynthesis in pear fruit, the *PbPFP* overexpression vector was injected into ‘Dangshansuli’ fruits at 35 DAB. Compared with the uninjected sites, the content of lignin increased significantly at the injection sites of 35S-*PbPFP*, and the lignin content at the injection sites of pCAMBIA-1300 did not change significantly (Fig. 10G). Lignin content by chemical analysis is consistent with the observation result (Fig. 10H). The lignin content in tobacco (*Nicotiana tabacum* L.) leaves significantly increased after the transient expression of *PbPFP* as well (Fig. 10J). By qRT-PCR analysis, the expression of *PbPFP* was increased at the injection sites of 35S-*PbPFP* in pear fruits and tobacco leaves (Fig. 10I, K).

## Discussion

The biosynthesis of lignin is an energy-consuming process with a large influx of carbon [46]. The content of carbon in lignin is higher than most other components in plants [32]. A lot of evidences show that glucose is an important carbon source for lignin biosynthesis [18], and glucose metabolism is closely related to the phenylpropanoid pathway [31, 32]. The effect of glucose on lignin biosynthesis was identified by using pear calli as material, and the biosynthesis of lignin in pear calli was significantly promoted by glucose. The application of exogenous glucose increased the influx of carbon in pear calli. Compared with the untreated control, the expression levels of many DEGs, which were involved in glucose metabolism, shikimic acid pathway, phenylpropanoid pathway and so on, were up-regulated in the pear calli treated with 4% glucose.

Primary metabolism plays an important role in secondary metabolism [37]. The increased expressions of genes in glycolysis are usually accompanied by activation of the pentose phosphate pathway, shikimate pathway and phenylpropanoid pathway [45]. HXK is an important enzyme in glycolysis and plays an important role in glucose sensing and signal transduction [47]. However, RNA-seq analysis revealed no difference in hexokinase expression, suggesting that HXK may not play an important role in the increased lignin biosynthesis due to glucose treatment. The sugar-mediated control of lignin deposition may occur through the hexokinase-independent sugar signaling pathway by treating *Arabidopsis* hypocotyls with different sugars [32], which is consistent with our results. HXK, PFK/PFP, and PK can simultaneously regulate glycolysis through rough and fine controls [34–36]. The regulation of carbon allocation caused by the influence of HXK, PFK/PFP, and PK on glycolysis might be the core that affects lignin biosynthesis, according to this. In present study, the most abundant DEGs in glycolysis were annotated as PFK/PFP, and five genes were identified as *PbPFK*, four of which (*Pbr028605.1*, *Pbr028606.1*, *Pbr028609.1*, and *Pbr028610.1*) were up-regulated while one (*Pbr008761.1*) was down-regulated, and only one gene was identified as *PbPFP* (*Pbr015118.1*) and was up-regulated. The expression pattern of *PbPFP* at early fruit developmental stages in ‘Dangshansuli’ fruits was consistent with the trend of lignin content, which indicated that *PbPFP* might be involved in the biosynthesis of lignin in pear fruit. After transient expression of *PbPFP* in ‘Dangshansuli’ fruits at 35 DAB and tobacco leaves, the lignin content at the injection sites increased significantly, indicating that *PbPFP* might play a positive role in lignin biosynthesis in response to glucose treatment. It might be the result of regulating glycolysis and thus affecting carbon flux.

Glucose is an important signal molecule [32, 48], and can induce phenylpropanoid compounds by up-regulating the expressions of genes related to the phenylpropanoid pathway [49]. Over-expressions of *PbPAL1* (*Pbr008363.1*) and *PbPAL2* (*Pbr008387.1*) increase the lignin biosynthesis in *Arabidopsis* stems [50]. *PbPAL1* responded to glucose stimulation in calli, and its expression was significantly up-regulated, while no significant changes was observed in *PbPAL2*. *PbHCT49* (*Pbr022422.1*) and *PbHCT65* (*Pbr036245.1*) were up-regulated by glucose. Among them, the expression pattern of *PbHCT49* is consistent with the changes in stone cell content during pear fruit development [51]. *PbCOMT3* (*Pbr040044.1*) was significantly differentially expressed and up-regulated more than 6-fold in treated pear calli. In addition, another four *PbCOMTs* (*Pbr040042.1*, *Pbr040045.1*, *Pbr025883.1*, and *Pbr007791.1*) were similarly up-regulated. *PbPRX53* (*Pbr026235.1*) is closely related to the change of lignin content in pear fruits [52]. *PbPRX8* (*Pbr000691.1*) plays an important role in the early stage of fruit development [53]. In the present study, the expressions of *PbPRX8* and *PbPRX55* were up-regulated by glucose, indicating that *PbPRX8* and *PbPRX55* might play an important role in the enhancement of lignin biosynthesis in response to glucose treatment.

Glucose also interacts with plant hormone signaling to regulate plant physiological metabolism [54]. The cross-action between glucose and ABA signaling is obvious, and glucose could induce the expression of genes involved in ABA biosynthesis, such as *ABA1*, *ABA3*, *ABI3*, *ABI5* and *AAO3* [25]. ABA positively regulates the expressions of lignin biosynthesis genes and promotes the synthesis of lignin [55]. In present study, 4 *ABF* genes (*Pbr017778.1*, *Pbr003516.1*, *Pbr007589.1* and *Pbr014592.1*) were up-regulated in response to glucose treatment (Fig. 5). It can be inferred that ABA TFs might regulate genes related to lignin biosynthesis. Furthermore, GA can significantly inhibit the enzyme activities of PAL and POD [44], and glucose plays a leading role in GA signaling by up-regulating *RGL2* [56]. DELLA protein is the main repressor of GA signaling [57]. The *PbDELLA* (*Pbr024549.1*) was up-regulated under glucose treatment, which might be one of the reasons for the increase of lignin content. The over-expressions of *PvGA2ox5* and *PvGA2ox9* in switchgrass reduced the lignin content of the transgenic lines, and the ratio of S/G lignin monomer was accompanied by a decrease in the expressions of lignin biosynthesis genes [58]. After RNA-seq analysis, 5 genes were identified as *GA2ox6*, 4 of which were down-regulated, which might be related to the increase of lignin content in pear calli after glucose treatment (Additional file 8: Table S5). Moreover, SA has been shown to increase the activity of PAL and positively regulate the accumulation of phenylpropanoids [59]. SA could combine with glucose to form SA-glucoside [60], which could induce the expression of the SA marker gene *PR-1* [61]. A total of 6 *PbPR-1* genes were identified, and their expression levels were all up-regulated by glucose, as was the expression of two *PbPAL* genes in the glucose-treated calli. The expressions of genes related to the phenylpropanoid pathway, especially the lignin biosynthesis pathway, should be affected by the cross-action of glucose and hormone signaling. However, which hormone signal is most affected by glucose treatment and how the cross-action affects lignin biosynthesis needs to be further explored.

The expressions of many TFs can also be induced by glucose. *MYB28*, *MYB29* and *MYB75* are induced by glucose to regulate the development of *Arabidopsis* [62, 63]. *OsMYBS1* and two homologous genes (*MYBS1* and *MYBS2*) are involved in seedling development in response to glucose [64, 65]. *AtbZIP1*, as a sugar-sensitive TF, was negatively regulated by glucose [66]. Some potential TFs regulated by glucose were identified in present study. The loss of functional *MYB103* results in a decrease in S-lignin content with an equivalent increase in G-lignin content [39]. *Pbr035306.1* and *Pbr020777.1*, as the homologous genes of *AtMYB103*, were down-regulated in the calli treated with glucose. It may be related to the fact that G-lignin is the main component of lignin in pear fruit [67]. *MYB52* could slightly induce the expression of genes in the secondary wall biosynthesis pathway [40]. *Pbr016851.1*, *Pbr002006.1* and *Pbr035515.1*, as the homologous genes of *AtMYB52*, were up-regulated in the calli treated with glucose. These genes might be involved in the glucose-mediated transcriptional regulation of the increase in lignin content in pear calli.

## Conclusions

Glucose metabolism is closely related to the phenylpropanoid pathway. However, the molecular basis of glucose regulating lignin biosynthesis in pear fruits and related genes has not been elucidated. In this study, exogenous glucose activated the glucose signaling pathway and modulated its downstream metabolic activities. 6566 DEGs were obtained by RNA-seq from glucose-treated pear calli. We have identified some candidate genes that might play important roles in glucose metabolism and lignin biosynthesis. Transient expression showed that *PbPFP* positively regulates lignin biosynthesis. Crosstalk of glucose signaling and hormone signaling, especially ABA, GA and SA signaling, and regulation of glucose-responsive TFs might play an important role in glucose-mediated lignin biosynthesis. This study is the first to use transcriptome analysis to investigate the regulatory role of glucose in lignin biosynthesis in stone cell formation in pear, providing a valuable resource for genetic research on the regulation of lignin biosynthesis. Future studies could focus on glucose-responsive transcription factors, and it would be very promising and meaningful to study how they sense glucose signals and regulate downstream phenylpropanoid pathway genes.

## Methods

### Plant materials and treatment

Pear calli derived from the flesh of young ‘Clapp’s Favorite (*P. communis*) fruit were darkly grown on the MS solid medium with sucrose (30 g/L), 6-benzylaminopurine (0.5 mg/L), and 2, 4-dichlorophenoxyacetic acid (1.0 mg/L) according to Bai et al. [68]. The calli were subcultured every half a month, then transferred to MS solid medium containing 6-benzylaminopurine (0.5 mg/L), 2, 4-dichlorophenoxyacetic acid (1.0 mg/L) and different concentrations of glucose (0, 1, 2, 3 and 4%) and the controlled pear calli were transferred to the MS solid medium containing 6-benzylaminopurine (0.5 mg/L), 2,4-dichlorophenoxyacetic acid (1.0 mg/L) with no glucose as blank control. Samples of pear calli were collected 15 days after treatment (with three biological replicates), immediately frozen in liquid nitrogen, and stored at − 80 °C until analyzed.

‘Dangshansuli’ fruits at 35 DAB were provided by Ziyinghe Orchard, Xiaji Town, Baoying County, Jiangsu Province, China. Tobacco (*Nicotiana tabacum* L.) was cultivated under short-day conditions and the temperature was controlled at 25 °C.

### Determination of lignin content

Pear calli were dried and ground into powder. 0.01 g sample (three replicates for each sample) was taken to determine lignin content by the acetyl bromide method according to Tao [5]. Samples were ground with 95% ethanol, then washed 3 times with 95% ethanol and ethanol: hexane (1:2, v/v), and dried. The dried precipitates were digested in 2 ml of 25% (v/v) acetyl bromoacetic acid solution and reacted at 70 °C for 30 min. The reaction was terminated by adding 0.9 ml of 2 M NaOH, and then 5 ml of acetic acid and 0.1 ml of 7.5 M hydroxylamine hydrochloride were added. The volume was adjusted to 10 mL with acetic acid, and the absorbance at A280 was determined. Finally, lignin content was calculated according to a lignin standard sample (Sigma-Aldrich, USA) curve.

### RNA extraction and RNA-seq library preparation, and sequencing

Total RNAs were extracted using a Plant RNA Isolation Kit (RNA Simply Total RNA Kit; Tiangen, Beijing, China) according to the manufacturer’s instructions. The degradation of RNA was monitored on 1% agarose gels, and the purity of RNA was checked using a NanoPhotometer®spectrophotometer (Implen, CA, USA). RNA concentrations were measured using a Qubit®RNA Assay Kit in a Qubit® 2.0 Fluorometer (Life Technologies, CA, USA). RNA integrity was evaluated using the RNA Nano 6000 Assay Kit of the Agilent Bioanalyzer 2100 system (Agilent Technologies, CA, USA).

RNA-seq library preparation and sequencing were performed by the Beijing Allwegene Technology (Beijing, China). RNA was enriched by magnetic beads with Oligo (dT). Then, the mRNA was broken into short fragments after the fragmentation buffer was added. Subsequently, six-base random primers (random hexamers) were used to reverse transcribe to synthesize one-strand cDNA using mRNA as a template, and then buffer, dNTPs and DNA polymerase I were added to synthesize two-strand cDNA. Next, AMPure XP beads were used to purify the double-stranded cDNA. The ends of the purified double-stranded cDNA were repaired, A and a linker were added. The double-stranded cDNA fragment size was selected by AMPure XP beads, and finally, PCR amplification was performed to construct a cDNA library. Then Agilent 2100 coupled with Illumina high-throughput sequencing platform (HiSeqTM2500/4000) was employed to check the library quality and sequence. The clean reads were mapped to the *Pyrus bretschneideri* genome sequence (http://gigadb.org/dataset/100083).

### DEGs analysis and functional annotation

In order to determine the difference in gene expressions between the glucose treatment and the control calli, the gene expression level was calculated based on the fragments per kilobase per million (FPKM) corresponding to the clean-read counts. BH (fdr correction with Benjamini/Hochberg) method was used to control the false discovery rate to adjust the *p*-value [69], and the DEGs with a *p*-value < 0.05 and |log_2_(fold change)| ≥ 1.5 were selected. Based on the pear genome database, all differentially expressed genes were annotated through gene ontology (GO; http://www.geneontology.org) and Kyoto Encyclopedia of Genes and Genomes (KEGG; http://www.genome.jp/kegg/) [70]. The GO annotations contain the biological processes, cellular components, and molecular functions of gene products. Each category is defined separately. KEGG is a comprehensive database that integrates genomic information, chemical information and biological system function information. All DEGs were aligned with NCBI non-redundant nucleotide sequences, NCBI non-redundant protein sequences (Nr), Swiss-Prot and Unipro protein databases.

### The analysis of transcription factor (TF)

We analyzed the DEGs determined by the annotated databases to mine for TFs using a Linux server. The data were further aligned with the protein sequences of the plant TF database PlantTFDB (version 3.0). Plant TFs were obtained from the TF database website (http://plntfdb.bio.uni-potsdam.de/v3.0/), and then analyzed the annotated TFs with an e-value ≤10^− 10^ using BLASTX. Finally, annotated transcription factors from pears involved in response to the glucose treatment were analyzed and classified into different groups based on the standards of the PlnTFDB (version 3.0).

### qRT-PCR validation

The coding region sequences of genes were downloaded from the pear genome, and Primer Premier 5 was used to design specific primers. The RNA samples used in the RNA-seq experiment were again used for qRT-PCR analysis. According to the manufacturer’s instructions, the first-strand cDNA was synthesized by one-step gDNA removal of transcripts and cDNA synthesis SuperMix (TRANGEN). The qRT-PCR used LightCycler480SYBR Green Master (Roche, USA) system. The reaction system was LightCycler480SYBR green I Master (Roche, Basel, Switzerland) for 10 μL, each primer was 200 nM, template cDNA was 100 ng, double distilled water was added to 20 μl. *Tubulin* (*Pbr028019.1*) was used as the reference gene, and the experiment was performed for three biological replicates. The PCR condition was 95 °C for 5 min, 55 cycles of 95 °C for 3 s, 60 °C for 10 s, 72 °C for 30 s, and the final extension was 72 °C for 3 min [71]. After the reaction, melt curve analysis was performed to ensure the specificity of the primer amplified fragment. Finally, the expression level of each sample was calculated using the 2^-△△CT^ method [72]. The qRT-PCR primers were listed in Additional file 9: Table S6.

### Transient expression of *PbPFP*

According to Xue et al. [73], pCAMBIA-1300 vector was subjected to double enzyme digestion to construct 35S-PbPFP for overexpression, pCAMBIA-1300 and 35S-PbPFP were transformed into *A. tumefaciens* (GV3101), inoculated on the LB solid medium containing Knan and RFP, cultivate at 28 °C until a single colony grows, dip a sterile toothpick into the LB liquid medium, cultivated at 28 °C, centrifuge and resuspend the bacterial solution to the buffer (10 mM MgCl_2_, 10 mM MES, 200 μM AS, pH 5.5), cultivate 3–6 h, use a needleless syringe to inject the bacteria containing the target gene vector into the pear fruits at 35 DAB and tobacco (*Nicotiana tabacum* L.) leaves. The small hole is close to the surface of the pear fruit, and the liquid is used to infiltrate the pear fruit with pressure to avoid mechanical damage. The fruits and tobacco are cultivated at 25 °C, first in the dark for 24 h, and then transferred to 16 h light, 8 h dark photoperiod for 5 d. Dying the pear longitudinally and determining the lignin content.

### Statistical analysis

All data were statistically analyzed with Microsoft Excel (Microsoft Corporation, Redmond, WA, USA) and SPSS 24 (SPSS Inc., Chicago, IL, USA), and graphed with GraphPad Prism 5. There was a significant difference between the treatment and the control (*p < 0.05, **p < 0.01, and ***p < 0.001).

## Supplementary Information

**Additional file 1: Figure S1.** Lignin content of calli treated with different concentrations of glucose. Different lowercase letters on the columns indicate that the treatment differs significantly at the 1% level.

**Additional file 2: Figure S2.** Heat map of transcription factor expressions. A: ERF, B: bHLH, bZIP, TCP, C: C2H2, ARF, HSF.

**Additional file 3: Figure S3.** Correlation analysis between RNA-Seq and qRT-PCR data.

**Additional file 4: Table S1.** Sequencing data statistics Q20 analysis.

**Additional file 5: Table S2.** Alignment of reads with reference sequence.

**Additional file 6: Table S3.** The DEGs were identified as PbPFK/PFP.

**Additional file 7: Table S4.** DEGs involved in plant hormone signal transduction pathways.

**Additional file 8: Table S5.** The DEGs were identified as PbGA2ox6.

**Additional file 9: Table S6.** Primers used for qRT-PCR analysis.

## Data Availability

The relevant data in this study are included in this article and the supplementary files. The plant materials involved in this study are kept in the life science building of Nanjing Agricultural University. The transcriptome datasets supporting the conclusions of this study have been uploaded to the National Center for Biotechnology Information (https://dataview.ncbi.nlm.nih.gov/object/PRJNA801923?reviewer=smt1v6atl5loa3qog9a58ud1hc).

## Abbreviations

4CL: 4-hydroxycinnamate CoA ligase
AAT: Aspartate aminotransferase
ABA: Abscisic acid
ABF: ABA Binding Factor
AHP: Histidine-containing phosphotransfer protein
ARF: Auxin response factor
AUX/IAA: Auxin-responsive protein IAA
AUX1: Auxin influx carrier
C3H: p-coumarate 3-hydroxylase
C4H: Cinnamate 4-hydroxylase
CCR: Cinnamoyl CoA reductase
CM: Chorismate mutase
COMT: Caffeic acid O-methyltransferase
CRE1: Cytokinin response 1
DEGs: Differentially expressed genes
DHQ: Dehydroquinate
E-4-P: 4-phosphate erythrose
ENO: Enolase
F5H: Ferulate 5-hydroxylase
FPKM: Fragments per Kilobase Million
GA: Gibberellin
GA2ox6: Gibberellin 2-oxidase
GO: Gene Ontology
HCT: Hydroxycinnamoyl transferase
KEGG: Kyoto Encyclopedia of Genes and Genomes
PAL: Phenylalanine ammonia-lyase
PEP: Phosphoenolpyruvate
PFK: 6-phosphofructokinase
PFP: pyrophosphate-fructose-6-phosphate-phosphotransferase
PK: pyruvate kinase
PP2C: Protein phosphatase 2C
PPP: Pentose phosphate pathway
PRX/POD: Peroxiredoxins
qRT-PCR: Quantitative real-time PCR
SK: Shikimate kinase
SnRK2: Serine/threonine-protein kinase
SOX: Sorbitol oxidase
TAT: Ttyrosine aminotransferase
TFs: Transcription factor
TIR1: Transport inhibitor response 1
DAB: Days after full bloom
