# Supplementary material for: Transcriptome analysis provides new ideas for studying the regulation of glucose-induced lignin biosynthesis in pear calli
Source: BMC Plant Biol. 2022 Jun 27;22:310. doi: 10.1186/s12870-022-03658-x (PMC9235211; doi:10.1186/s12870-022-03658-x)
Supplement: Supplementary file 1 — Additional file 1: Figure S1. Lignin content of calli treated with different concentrations of glucose. Different lowercase letters on the columns indicate that the treatment differs significantly at the 1% level. [file 12870_2022_3658_MOESM1_ESM.pdf]

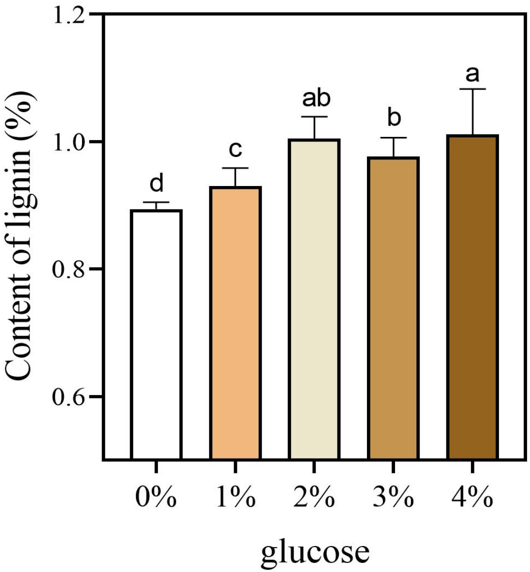

Additional file 1: Figure S1. Lignin content of calli treated with different concentrations of glucose. Different lowercase letters on the columns indicate that the treatment differs significantly at the 1% level.
