# Supplementary material for: Transcriptome analysis provides new ideas for studying the regulation of glucose-induced lignin biosynthesis in pear calli
Source: BMC Plant Biol. 2022 Jun 27;22:310. doi: 10.1186/s12870-022-03658-x (PMC9235211; doi:10.1186/s12870-022-03658-x)
Supplement: Supplementary file 2 — Additional file 2: Figure S2. Heat map of transcription factor expressions. A: ERF, B: bHLH, bZIP, TCP, C: C2H2, ARF, HSF. [file 12870_2022_3658_MOESM2_ESM.pdf]

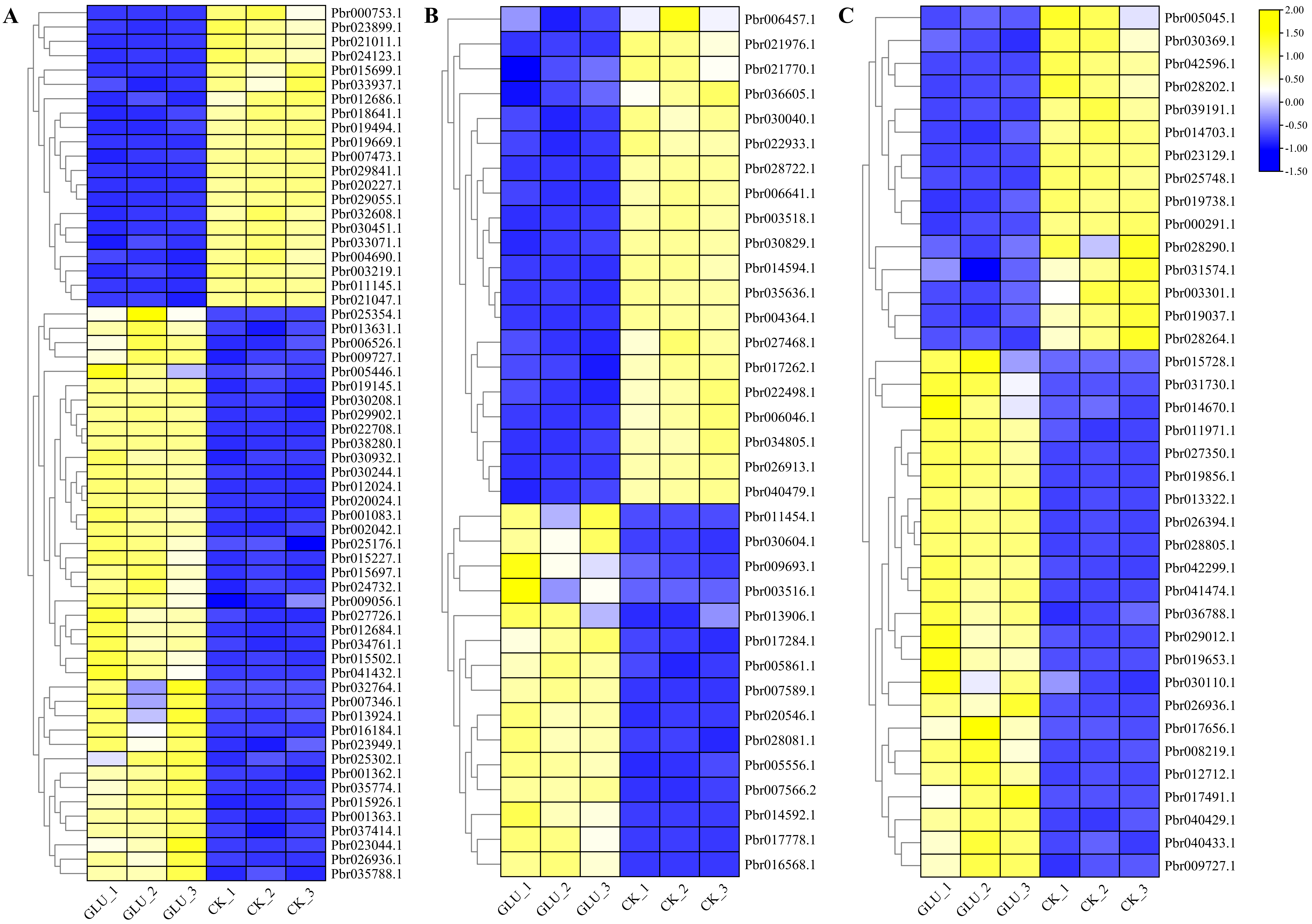

Additional file 2: Figure S2. Heat map of transcription factor expressions. A: ERF, B: bHLH, bZIP, TCP, C: C2H2, ARF, HSF.
