# Supplementary figures and images for: Transcriptome analysis provides new ideas for studying the regulation of glucose-induced lignin biosynthesis in pear calli
Source: BMC Plant Biol. 2022 Jun 27;22:310. doi: 10.1186/s12870-022-03658-x (PMC9235211; doi:10.1186/s12870-022-03658-x)

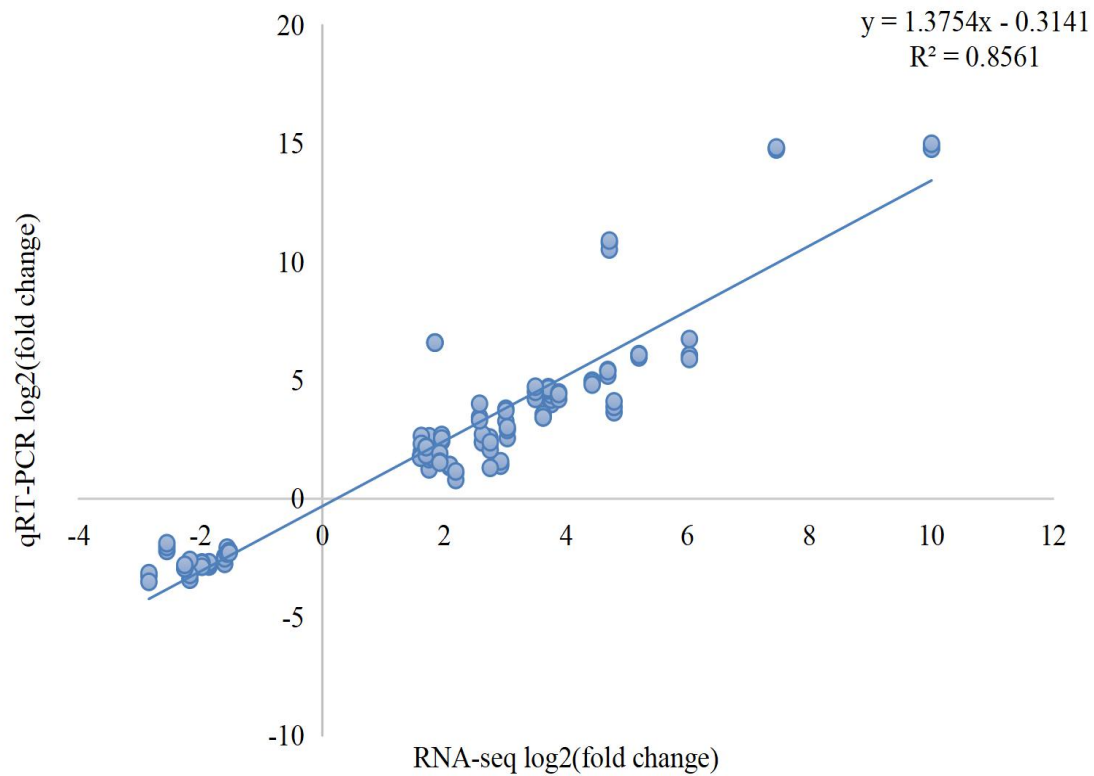

**Additional file 3: Figure S3.** Correlation analysis between RNA-Seq and qRT-PCR data.

Supplement: Supplementary file 3 — Additional file 3: Figure S3. Correlation analysis between RNA-Seq and qRT-PCR data. [file 12870_2022_3658_MOESM3_ESM.pdf]
